# Periostin Accelerates Bone Healing Mediated by Human Mesenchymal Stem Cell-Embedded Hydroxyapatite/Tricalcium Phosphate Scaffold

**DOI:** 10.1371/journal.pone.0116698

**Published:** 2015-03-16

**Authors:** Soon Chul Heo, Won Chul Shin, Mi Jeong Lee, Ba Reun Kim, Il Ho Jang, Eun-Jung Choi, Jung Sub Lee, Jae Ho Kim

**Affiliations:** 1 Department of Physiology, School of Medicine, Pusan National University, Yangsan 626-870, Gyeongsangnam-do, Republic of Korea; 2 Department of Orthopaedic Surgery, School of Medicine, Pusan National University, Yangsan 626-870, Gyeongsangnam-do, Republic of Korea; 3 Research Institute of Convergence Biomedical Science and Technology, Pusan National University Yangsan Hospital, Yangsan 626-770, Gyeongsangnam-do, Republic of Korea; University of Pittsburgh, UNITED STATES

## Abstract

**Background:**

Periostin, an extracellular matrix protein, is expressed in bone, more specifically, the periosteum and periodontal ligaments, and plays a key role in formation and metabolism of bone tissues. Human adipose tissue-derived mesenchymal stem cells (hASCs) have been reported to differentiate into osteoblasts and stimulate bone repair. However, the role of periostin in hASC-mediated bone healing has not been clarified. In the current study, we examined the effect of periostin on bone healing capacity of hASCs in a critical size calvarial defect model.

**Methods and Results:**

Recombinant periostin protein stimulated migration, adhesion, and proliferation of hASCs *in vitro*. Implantation of either hASCs or periostin resulted in slight, but not significant, stimulation of bone healing, whereas co-implantation of hASCs together with periostin further potentiated bone healing. In addition, the number of Ki67-positive proliferating cells was significantly increased in calvarial defects by co-implantation of both hASCs and periostin. Consistently, proliferation of administered hASCs was stimulated by co-implantation with periostin *in vivo*. In addition, co-delivery of hASCs with periostin resulted in markedly increased numbers of CD31-positive endothelial cells and α-SMA-positive arterioles in calvarial defects.

**Conclusions:**

These results suggest that recombinant periostin potentiates hASC-mediated bone healing by stimulating proliferation of transplanted hASCs and angiogenesis in calvarial defects.

## Introduction

Restoration of extended bony defects due to traumatic injury, tumor resection, or genetic disorders is a challenging problem in reconstructive surgery [1]. Autologous bone grafting is believed to be an effective method and is regarded as the “gold standard” for the clinical therapies of critical-sized bone defects. Major problems over the use of autologous free tissue transfer, however, include limited tissue supplies and significant donor-site morbidity, such as hemorrhage, infection, and chronic pain [2–4]. Among cells with therapeutic potential, mesenchymal stem cells (MSCs) have become a promising therapeutic cell source that addresses the limitations of autologous cell-based tissue engineering for bone reconstruction [5], due to their capacity for self-renewal and their ability to differentiate into several lineages of mesenchymal tissue, including adipose, cartilage, muscle, and bone [6].

Bone marrow-derived MSCs (BMSCs) are the most commonly used cell source for ongoing clinical applications. However, regarding the disadvantages related to the use of BMSCs, including patients suffering from isolation and low cell yield, adipose tissue-derived MSCs (ASCs), which can be obtained in large quantities and yield more MSCs compared to BMSCs, are an alternative source of MSCs [7, 8]. Studies in several animal models have demonstrated that implantation of ASCs can improve bone formation. For example, human ASCs (hASCs) seeded on demineralized bone matrix (DBM) implanted in abdominal muscles of rats showed 30% higher bone formation compared to empty scaffolds [9]. In addition, enhanced repairs in rat or mouse cranial defects following implantation of human ASCs seeded on DBM [10] or hydroxyapatite (HA) coated poly(-lactic-co-glycolic acid) [11] have been reported. In contrast, however, *in vivo* studies have shown inconsistent results of stem cell-based bone formation, such as only small amounts of bone, presence of dystrophic calcification, and limited survival and engraftment of transplanted cells in the implanted host tissues [12–15]. Therefore, it is necessary to improve the efficiency of survival and engraftment of transplanted hASCs in defected bone tissues.

Periostin, originally known as osteoblast-specific factor, is a 93-kDa matricellular glutamate-containing protein, which is highly expressed during ontogenesis and in adult connective tissues, including periosteum [16], periodontal ligaments [17], and tendon [18]. Periostin has been reported to promote cell mobility, adhesion, and survival in a number of cell types [19, 20]. Periostin was reported to bind to integrin αvβ3 and αvβ5 and regulate cell adhesion and mobility *via* the Akt/protein kinase B pathway [21, 22]. During fetal development, multiple variants of periostin are preferentially expressed in the periosteum at a high level [23, 24] and detected in MSCs [16, 25–27]. Periostin has been reported to play a critical role in bone metabolism and bone formation [28]. It plays a key role in morphogenesis, postnatal development, and maintenance of bone tissues, including tooth [29]. Periostin deficiency led to increased bone damage and impaired injury response to fatigue loading in adult mice [30]. In periostin-deficient mice, collagen fibrillogenesis was disrupted in the periosteum [31] and studies on osteoblasts isolated from calvaria of these mice suggest a role in extracellular matrix organization [32]. In addition to the role of periostin in bone formation, intramuscular injection of recombinant periostin protein resulted in stimulation of angiogenesis and attenuation of severe limb loss in a murine model of limb ischemia [33]. These results suggest that periostin plays a key role in tissue repair beyond its role as a structural protein. However, the role of periostin in stem cell-mediated bone repair has not yet been clarified.

The aim of this study was to improve stem cell-based bone regeneration in a murine critical-sized calvarial bone defect model using periostin. We examined the effects of periostin on bone formation and proliferation of exogenously transplanted human ASCs *in vivo*.

## Materials and Methods

### Materials

α-minimum essential medium, trypsin, Hank’s balanced salt solution (HBSS), and fetal bovine serum were purchased from Invitrogen (Carlsbad, CA). Culture plates were purchased from Nunc (Roskilde, Denmark). A recombinant human periostin protein (catalog number, 3548-F2) was purchased from R&D Systems, Inc. (Minneapolis, MN) and lyophilized protein was reconstituted in sterile PBS at a concentration of 100 μg/mL. HyStem hydrogel scaffold (HYS020) were purchased from Sigma (St. Louis, MO) which are composed of HyStem and Extralink. Anti-Ki67 polyclonal antibody (NCL-ki67p) was purchased from Novocastra Laboratories (Newcastle, UK). Anti-PCNA polyclonal antibody (sc-7907) was purchased from Santa Cruz Biotechnology, Inc. (Santa Cruz, CA).

### Cell culture

hASCs were isolated from subcutaneous adipose tissues of five female patients under the age of 40. Subcutaneous adipose tissue was obtained from elective surgeries with the patient’s written informed consent and this study was approved by the Institutional Review Board of Pusan National University Hospital. For isolation of hASCs, adipose tissues were treated with collagenase type I suspension (1 g/L of Hank’s balanced salt solution with 1% bovine serum albumin) for 60 min at 37°C. The floating adipocytes were separated from the stromal-vascular fraction by centrifugation. The cell pellet was resuspended in α-minimum essential medium supplemented with 10% FBS, 100 U/mL penicillin, and 100 μg/mL streptomycin, and cells were plated in tissue culture dishes at a density of 3500 cells/cm^2^. The primary hASCs were cultured for 4–5 days until they reached confluence (defined as passage 0). hASCs used in these experiments were passaged 2–5 times. The hADSCs were positive for CD29, CD44, CD73, CD90, and CD105, all of which have been reported to be mesenchymal stem cells marker proteins. However, these cells did not express CD31, CD34, and CD45, which are markers for endothelial cells, hematopoietic progenitor cells, and leukocytes, respectively (data not shown).

### Cell migration assay

Migration of hASCs was assayed using a disposable 96-well chemotaxis chamber (Neuro Probe, Inc., Gaithersburg, MD). Checkerboard analysis was performed as follows for determination of whether periostin-induced cell migration represented chemotaxis (directed migration) or chemokinesis (random migration). Membrane filters (8-μm pore size) in disposable 96-well chemotaxis chambers (Neuro Probe, Gaithersburg, MD) were pre-coated overnight with 20 μg/mL rat-tail collagen at room temperature. Briefly, hASCs were resuspended in α-MEM in the absence or presence of periostin just before they were transferred to the upper chamber and serum-free α-MEM in the absence or presence of periostin was added in the lower chamber for generation of concentration gradients of periostin between the upper and lower compartments. After incubation of the cells for 12 h at 37°C, the filters were disassembled. The number of cells that had migrated to the lower surface of each filter was determined by counting the cells under microscopy after staining with Hoechst.

### Cell adhesion assay

Ninety-six-well microculture plates (Falcon, Becton-Dickinson, Mountain View, CA) were incubated with recombinant periostin proteins at 37°C for 1 h, followed by blocking with HBSS containing 0.2% BSA for 1 h at 37°C. Cells were trypsinized and suspended in the culture media at a density of 2 × 10^4^ cells/well and were then added to each well of the plate. After incubation for 1 h at 37°C, unattached cells were removed by washing twice with HBSS. The number of attached cells was determined by counting the cells under microscopy at 100× magnification after staining with hematoxylin and eosin and expressed as the number of cells/high power field.

### Cell proliferation assay

To explore the effects of recombinant periostin on cell proliferation of hASCs, a colorimetric 3-(4,5-dimethylthiazol-2-yl)-2,5-diphenyltetrazolium bromide (MTT) assay was used. Cells were seeded in a 24-well culture plate at a density of 1×10^4^ cells/well, cultured for 48 h in serum-free α-MEM containing increasing amounts of periostin. Cells were washed twice with HBSS and incubated with 200 μL of MTT (0.5 mg/mL) for 2 h at 37°C. Formazan granules generated by the cells were dissolved in 100 μL of dimethylsulfoxide, and the absorbance of the solution at 562 nm was determined using a PowerWavex microplate spectrophotometer (Bio-Tek Instruments, Inc.; Winooski, VT) after dilution to a linear range and expressed as the relative percentage of control.

### Calvarial bone defect model

The animal experiments protocol was reviewed and approved by the Pusan National University Institutional Animal Use and Care Committee. BALB/CA-nu/nu (male, age 8–10 wks, weighing 22–24 g) were anesthetized with an intraperitoneal injection of 400 mg/kg 2,2,2-tribromoethanol (Avertin; Sigma) for a surgical operation. Full-thickness skin of BALB/CA-nu/nu mice was cut and the periosteum was elevated to expose the calvarial bone surface. Oversized bone defects (7 × 8 mm) were established on calvarial bones using a hand drill and a trephine bit. During surgery, sterile saline was dripped over the drilling area to protect from extensive heat damage. For transplantation of hASCs, approximately 1 × 10^6^ hASCs were mixed with 150 μL of HyStem hydrogel (Sigma-Aldrich) in the absence or presence of recombinant periostin protein (200 μg), followed by addition of 50 μL of Extralink (polyethylene glycol diacrylate) for gelation of the hydrogel mixtures. The final hydrogel solution was immediately mixed with hydroxyapatite/tricalcium phosphate (HA/TCP) scaffold (Zimmer Inc.) particles (40 mg per a defect) as a carrier and then implanted into the calvarial bone defects. Prior to implantation, hASCs were labeled with the long-lasting cell tracker CM-DiI (Molecular Probes, Eugene, OR) according to the manufacturer’s instructions. Animals were divided into 5 experimental groups: group 1, hydrogel + HBSS; group 2, hydrogel + HA/TCP; group 3, hydrogel + HA/TCP + hASCs; group 4, hydrogel + HA/TCP + periostin; group 5, hydrogel + HA/TCP + hASCs + periostin. The periosteum and skin were completely closed with non-absorbable sutures and calvaria was analyzed with micro-CT imaging for measurement of initial gap of calvarial defects. At 2 weeks or 8 weeks after transplantation, mice were euthanized with CO_2_ inhalation, and calvaria was dissected for micro-CT imaging, histochemistry, and immunostaining.

### Micro-CT analysis

To image bone formation in situ, micro computed tomography were performed using high resolution micro-CT imaging system (NFR Polaris-G90, NanoFocus Ray Co., Iksan, Korea). The following settings were used: an X-ray voltage of 70 kVp, current of 180 μA and an exposure time of 700 milliseconds for each of the 360 rotational steps. The reconstruction image size was 1,024 × 1,024 pixels, and the number of slices was 512. Three dimension images were used to reconstruct tomograms with a RayWare TM (NanoFocus Ray Co., Iksan, Korea). The coronal section of micro-CT imaging through the midlines of defects was selected for measurement of gap of defects. The gap of defects was quantified by measurement of distance between the advancing edges from coronal sectional micro-CT images of calvarial defects by NIH ImageJ software, followed by calculation of the percentages of gap of defects at week 8 to initial gap of defects.

### Histological and immunochemical analysis

For histological and immunochemical analysis, calvarial bone specimens were fixed with 4% paraformaldehyde for 24 h at 4°C and decalcified with 10% EDTA (pH 7.4) for 21 days before embedding in paraffin, sectioning, and staining with Massons’s trichrome or hematoxylin and eosin. For the quantification of the bone volume, H&E stained sections of three standardized locations within the defect were used. Traced the newly formed bone area within the defect and bone volume area at each section was calculated using NIH ImageJ software. For the quantification of the gap of defect which was defined as the distance between the advancing edges of newly formed bone, was measured in three serial sections of Massons’s trichrome stained images using ImageJ software. For staining of proliferating cells, the specimens were incubated with anti-Ki67 antibody, followed by biotinylated anti-rat IgG (Vector Laboratories, Burlingame, CA) and staining was visualized using biotin-avidin-peroxidase complexes (Vector Laboratories) and diaminobenzidine (Vector Laboratories). Slides were counter-stained with hematoxylin to enable visualization of nuclei. Under light microscopy, four staining sections were randomly photographed using a model DFC300FX mounted digital camera (Leica, Solms, Germany) in the calvarial defect site. Ki67-positive nuclei and total nuclei were counted using Image J software for measurement and represented as relative percentage of Ki67-positive cells per number of nuclei in each section. For immunofluorescence staining of PCNA, sections were incubated with anti-PCNA antibody for 2 h, followed by incubation with Alexa Fluor 488-conjugated secondary antibody (Invitrogen). The percentage of PCNA-positive nuclei/CM-DiI-positive nuclei per HPF was determined. Endothelial cells and smooth muscle cells were immunostained with rabbit anti-CD31 and rabbit anti-α-SMA antibodies, followed by incubation with Alexa 488 goat anti-rabbit or Alexa 568 goat anti-rabbit secondary antibodies. The specimens were finally washed and mounted in Vectashield medium (Vector Laboratories) with 4',6-diamidino-2-phenylindole for visualization of nuclei. Fluorescence images were collected using a Leica TCL-SP2 confocal microscope system (Leica Microsystems Heidelberg GmbH, Heidelberg, Germany). Capillary density and the number of arterioles/arteries were determined by counting the number of CD31-positive and α-SMA-positive features per high power field (×400). The numbers of PCNA-, CD31-, and α-SMA-positive features were quantified by two independent observers who were blinded to the experimental conditions in four randomly chosen microscopic fields in each specimen.

### Statistical analysis

Results of multiple observations are presented as mean ± S.D. For analysis of multivariate data, group differences were assessed using one-way or two-way ANOVA, followed by Scheffé’s post hoc test.

## Results

### Periostin stimulates migration, adhesion, and proliferation of hASCs

**Fig 1:**
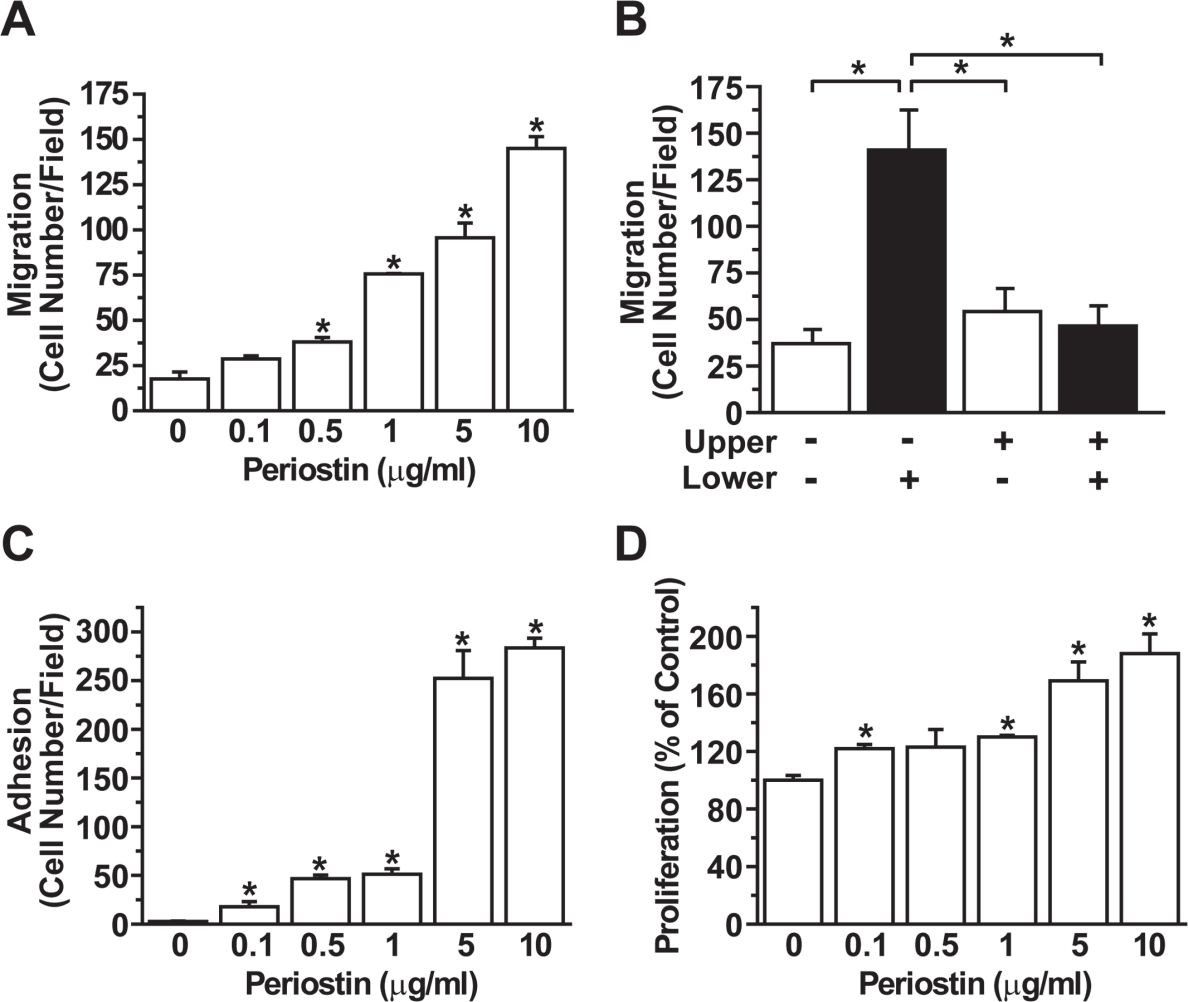
Periostin stimulates chemotaxis migration, adhesion, and proliferation activity of hASCs *in vitro*. (A) Dose-dependent effect of periostin on hASC migration. hASCs were loaded in the upper chamber of a chemotaxis chamber apparatus and recombinant periostin with the indicated concentration was placed in the lower chamber, followed by measurement of the number of migrated cells after 12 h incubation. (B) Checkerboard analysis of the periostin-induced migration of hASCs was measured using a chemotaxis chamber. Periostin was placed in either the bottom, top, or both chambers of the chemotaxis system and hASCs were loaded into the upper chamber. The number of migrated cells was quantified after incubation of the cells for 12 h. Data represent mean ± S.D.; *, p < 0.05. (C) 96-well plates were coated with indicated concentration of periostin and adhesion of hASCs onto the plates was determined. (D) Dose dependence of periostin-stimulated proliferation. hASCs were treated with the increasing concentrations of periostin for 3 days and proliferation of cells was determined by MTT assay. Data represent mean ± S.D. *, p < 0.05 vs control.

To explore the question of whether periostin can regulate migrating capability of hASCs, the effects of recombinant periostin on cell migration were determined using a chemotaxis chamber system. As shown in Fig. 1A, recombinant periostin dose-dependently stimulated migration of hASCs with a maximal stimulation at 10 μg/mL concentration. To elucidate whether the periostin-induced cell migration was chemotaxis or chemokinesis, checkerboard analysis was performed. Periostin-induced migration was only observed in the presence of a positive concentration gradient between two compartments (higher concentration in the lower chamber); migration did not occur with equal concentrations of periostin in the upper and lower chamber or with a negative gradient (higher concentration in the upper chamber), suggesting that periostin promotes chemotactic migration of hASCs (Fig. 1B). To evaluate the effect of periostin on adhesion of hASCs, adhesive capacities of hASCs on periostin-coated plates were determined. As shown in Fig. 1C, the number of adherent hASCs showed a gradual increase with increasing concentration of periostin coating. To determine whether periostin can affect proliferative ability of hASCs, cells were incubated with the increasing dose of periostin and proliferation was measured by MTT assay. As shown in Fig. 1D, recombinant periostin stimulated proliferation of hASCs in a dose dependent manner. Taken together, these results suggest that periostin plays a key role in chemotactic migration, adhesion, and proliferation of hASCs.

### Periostin stimulates hASC-mediated bone healing in a calvarial bone defect animal model

**Fig 2:**
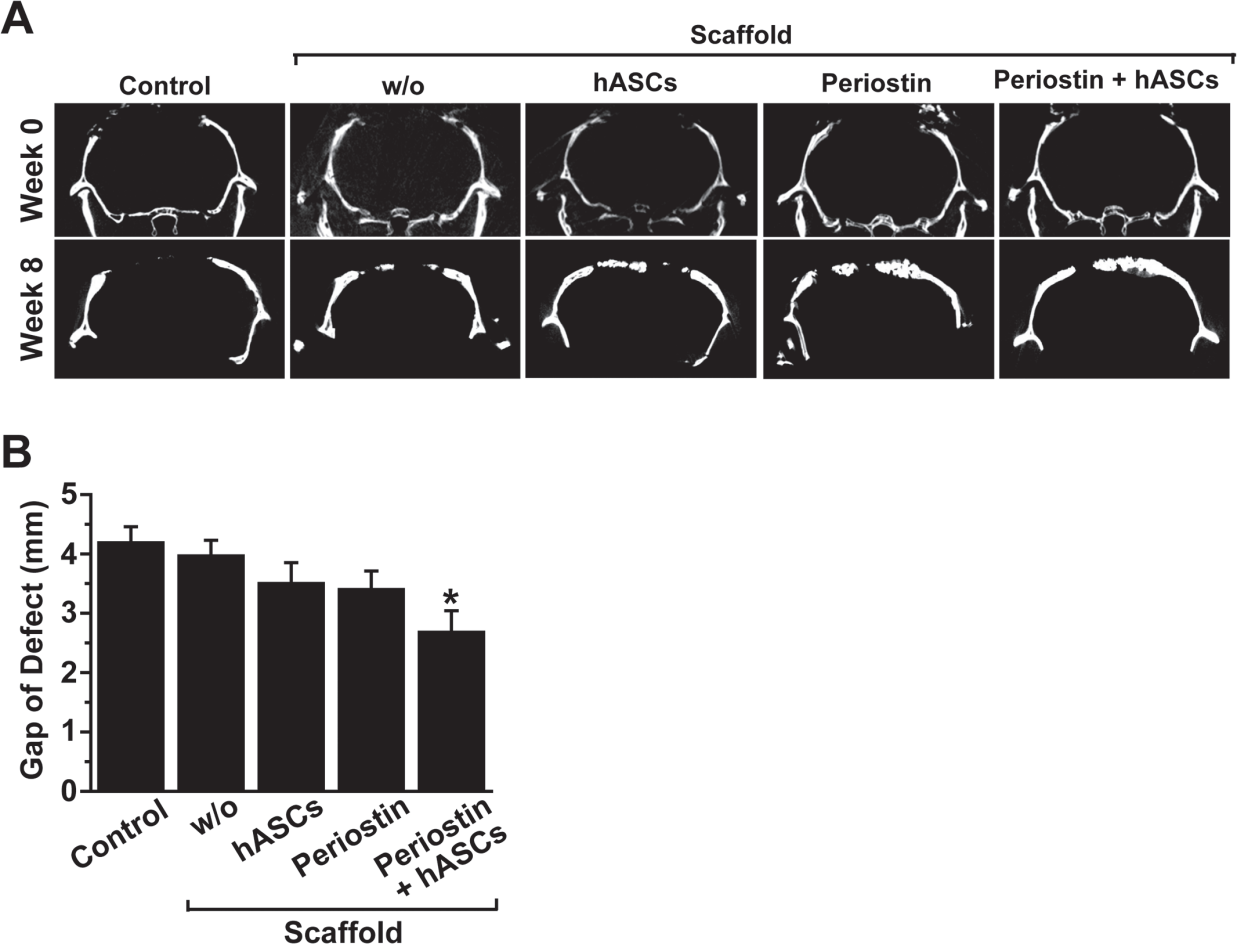
Micro-CT analysis of bone regeneration following implantation of hASCs and/or periostin into calvarial defects. Calvarial defects were implanted with HA/TCP scaffold bearing hASCs and/or periostin, or mock-treated (control). (A) Posterior view of a coronal sliced micro-CT images of the calvaria was captured at week 0 and 8. (B) The distance between the advancing edges was quantified from coronal-section view images of calvarial defects by Micro-CT. Data represent mean ± S.D. (n = 8). *, p < 0.05 vs control.

**Fig 3:**
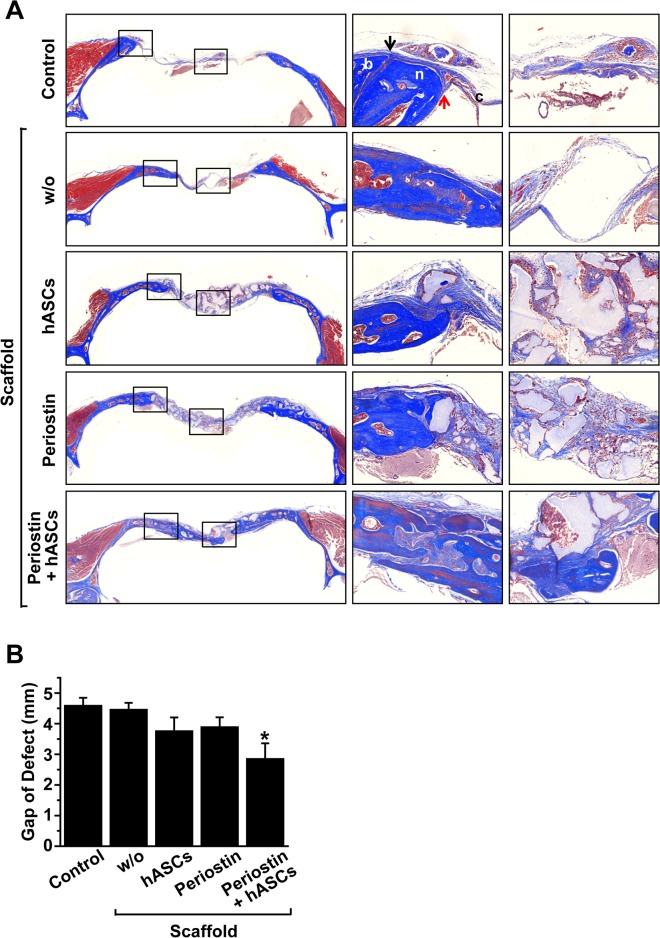
Effects of hASCs and/or periostin on regeneration of calvarial bone defects. (A) Masson’s trichrome staining of calvarial defects (blue color: mineralized bone tissue; red color: non-mineralized bone tissue). Higher magnification images of the regions highlighted by the black box in left panels are shown in right panels. (B) The gap of calvarial defects in the Massons’s trichrome staining images was quantified and shown as the distance between the advancing edges. Data represent mean ± S.D. (n = 8). *, p < 0.05 vs control.

Because periostin treatment stimulates migration, adhesion, and proliferation of hASCs *in vitro*, we next explored the question of whether periostin treatment might improve the efficiency of hASCs-based bone repair using an *in vivo* murine critical-sized calvarial bone defect model. Bone defects were generated in the calvarial bones using a trephine bit and a hand drill, and we implanted hASCs alone or together with periostin, or various controls, into the defective region. To enhance engraftment and cell differentiation of hASCs in bone defects, hASCs and periostin were mixed with HA/TCP scaffold, followed by implantation into calvarial defects. Analysis of cross-sectional images by micro-CT scanning showed that implantation of hASCs with periostin resulted in a significant reduction of the defective gap, which was quantified as the distance between the advancing edges, compared with the control (Fig. 2A, B). Implantation of hASC alone or periostin alone resulted in a slight, but not significant reduction of the gap. To further confirm the effects of hASCs and periostin implantation on repair of calvarial bone defect, critical sized calvarial defects were subjected to Masson's trichrome staining. Co-implantation of hASCs together with periostin increased the newly formed connective tissues, which were stained as blue color with Masson's trichrome stain (Fig. 3A). In addition, co-implantation of hASCs with periostin decreased the gap of defects that were deficient for connective tissues in calvarial bone (Fig. 3B).

**Fig 4:**
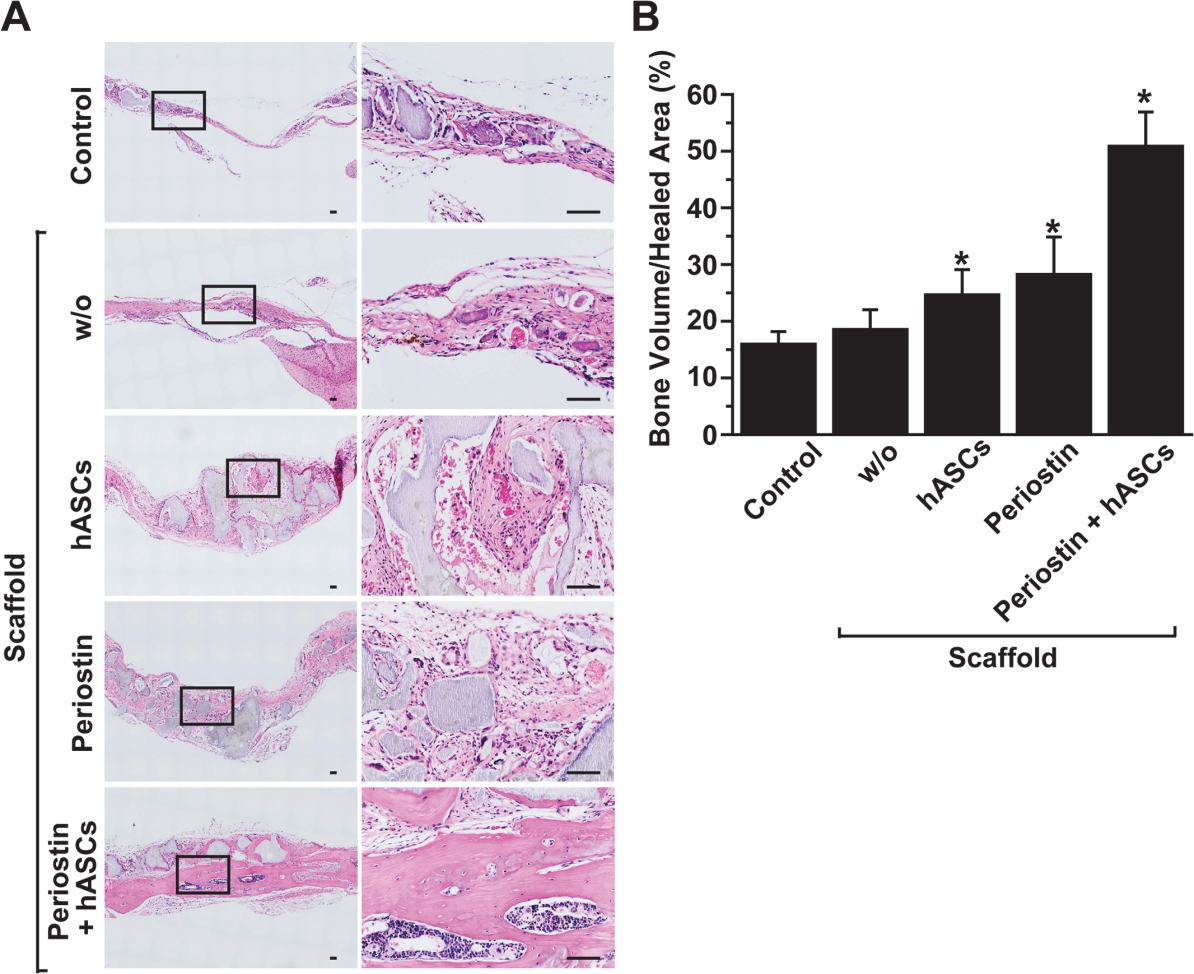
Histological analysis of newly regenerated bone after implantation of hASCs and/or periostin. (A) H&E staining of calvarial defects eight weeks after implantation of hASCs and/or periostin. Higher magnification images of the regions highlighted by the black box in left panels are shown in right panels. Scale bar = 100 μm. (B) Area of newly regenerated bone tissues was determined in the defected bone and the percentage of bone area per healed area was calculated. Data represent mean ± S.D. (n = 8). *, p < 0.05 vs control.

Next we performed H&E staining to confirm the result showing that co-implantation of hASCs and periostin stimulated bone repair. Implantation of either hASCs or periostin resulted in slightly increased volume of newly formed bone and co-implantation of hASCs with periostin much potently stimulated bone formation compared with the experimental groups in which either hASCs or periostin was transplanted (Fig. 4A, B). These results suggest that periostin cooperates with hASCs to enhance the wound healing of calvarial defects.

### Periostin stimulates proliferation of transplanted hASCs in defected bone

**Fig 5:**
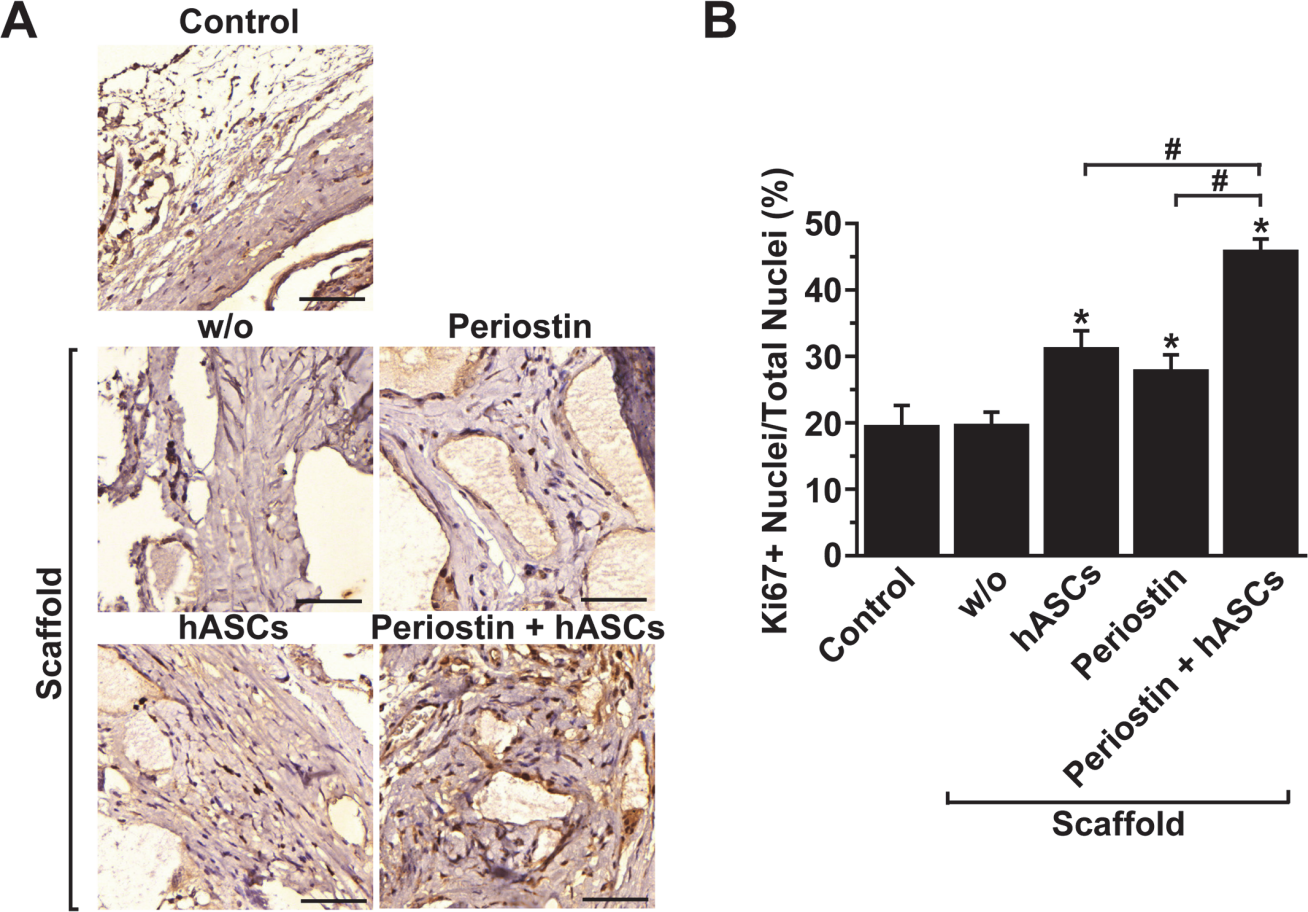
Effects of hASCs and periostin implantation on cell proliferation within defective calvarial bone. (A) Proliferating cells within defective calvaria were detected by immunostaining with anti-Ki67 antibody at week 8 after implantation of hASCs and/or periostin. Scale bar = 50 μm. (B) Numbers of Ki67-positive cells and total nuclei per field were counted and expressed as the relative percentage of Ki67-positive cells per total nuclei. Data represent mean ± S.D. (n = 8). #, p < 0.05; *, p < 0.05 vs control.

To explore the question of whether the bone repair stimulated by co-implantation of periostin and hASCs is mediated by increased proliferation within defected bone tissues, we determined the number of cells expressing Ki67, a proliferation marker, by immunostaining (Fig. 5A). The number of Ki67-positive cells showed a slight increase after implantation of hASCs or periostin alone compared with the control group. Combined implantation of hASCs and periostin further increased the number of Ki67-positive cells compared with the experimental groups transplanted with either hASCs or periostin (Fig. 5B). To elucidate the nature of Ki67-positive cells, we carried out double immunofluorescence staining with antibodies against Ki67 and osteocytes marker osteocalcin or pan-leukocyte marker CD45. Confocal microscopy images of immunofluorescence staining showed that both osteocalcin-positive osteocytes and CD45-positive leukocytes were positive for Ki67 staining (S1 Fig.). These results suggest that co-implantation of hASCs and periostin stimulates cell proliferation of osteocytes as well as leukocytes within calvarial defects.

**Fig 6:**
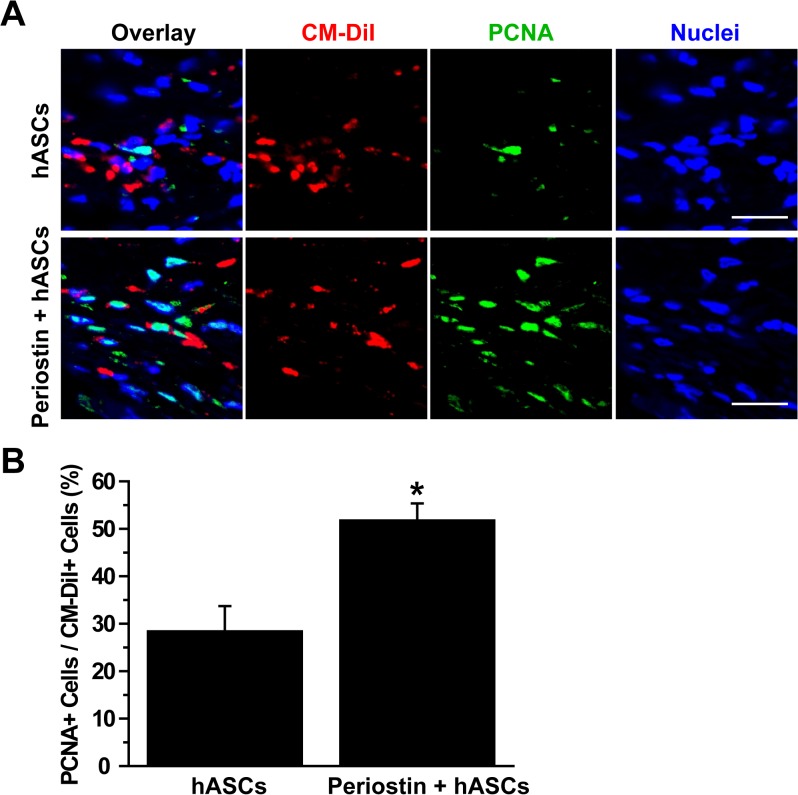
Effects of periostin on proliferation of implanted hASCs *in vivo*. (A) hASCs were labeled with CM-DiI and loaded into HA/TCP scaffold with or without periostin, followed by implantation of the scaffold into the defective calvaria. Two weeks after implantation, tissue specimens were immunostained with anti-PCNA antibody. Overlaid images of CM-DiI-positive hASCs (red color), nuclei (blue color), and PCNA (green color) are shown. Scale bar = 20 μm. (B) The numbers of PCNA- and CM-DiI-double positive cells, which indicate proliferating hASCs, were counted and the percentage of PCNA-positive cells per CM-DiI-positive cells was determined. Data represent mean ± S.D. (n = 8). *, p < 0.05 vs control.

Because periostin increased proliferation of hASCs *in vitro*, as shown in Fig. 1D, we attempted to determine whether periostin can increase proliferation of transplanted hASCs during bone repair. For tracing transplanted cells, hASCs were labeled with CM-DiI, followed by implantation of the CM-DiI-labeled cells into the defected calvaria in the presence or absence of periostin. Two weeks after implantation, the number of CM-DiI-positive cells expressing PCNA, a proliferation marker, in the defected calvaria was quantified by immunofluorescence staining. As shown in Fig. 6A, cells positive for both CM-DiI and PCNA were detected in the defective area. The percentage of PCNA-positive cells per CM-DiI-positive cells was significantly increased in the experimental group transplanted with hASCs and periostin compared with the control group transplanted with hASC alone (Fig. 6B). Taken together, these results suggest that periostin improves proliferation and survival of implanted hASCs and promotes the repair of defective calvaria.

### Co-implantation of hASCs and periostin stimulates angiogenesis in defected bone

**Fig 7:**
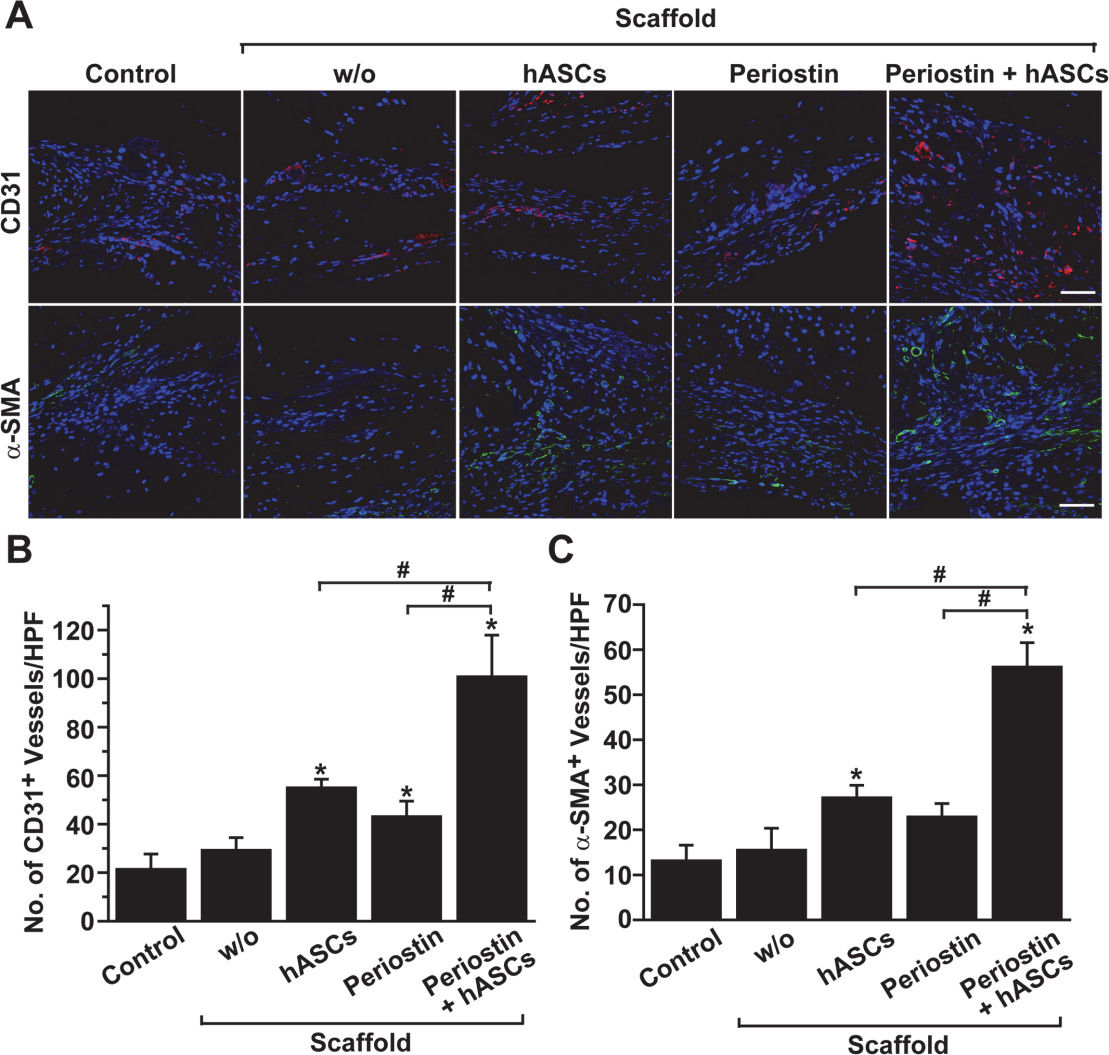
Effects of hASCs and periostin on angiogenesis in calvarial defects. (A) Immunostaining of endothelial cells and smooth muscle cells in calvarial defects implanted with hASCs and/or periostin. The specimens were immunostained with anti-CD31 (red color) or anti-α-SMA (green color) antibodies, and overlaid images with nuclei (blue color) are shown. Scale bar = 50 μm. The numbers of CD31-positive capillaries (B) and α-SMA-positive blood vessels (C) per high power field were counted. Data represent mean ± S.D. (n = 8). #, p < 0.05; *, p < 0.05 vs control.

Both hASCs and periostin have been reported to accelerate repair of ischemic tissues by stimulating angiogenesis *in vivo* [33, 34]. To explore the question of whether co-implantation of hASCs with periostin can affect angiogenesis in defected bone tissues, we quantified the numbers of CD31-positive capillaries and α-SMA-positive arterioles in calvarial defects after implantation of hASCs and/or periostin. As shown in Fig. 7A and 7B, implantation of hASCs caused an increase in the number of CD31-positive capillaries in calvarial defects. Administration of periostin caused a slight increase in the number of CD31-positive capillaries, and co-implantation of hASCs together with periostin further augmented the formation of capillaries in defected bone. In addition, implantation of hASCs caused an increase in the number of α-SMA-positive arterioles (Fig. 7A and 7C). Application of periostin resulted in a slight, but not significant increase in the number of α-SMA-positive arterioles. However, co-implantation of hASCs and periostin caused significantly up-regulated formation of α-SMA-positive arterioles in defected bone. These results suggest that periostin stimulated hASC-mediated angiogenesis in calvarial defects.

## Discussion

In the current study, we demonstrated that periostin promoted chemotaxis, adhesion, and proliferation of hASCs *in vitro* and co-delivery of hASCs with recombinant periostin promoted survival and proliferation of transplanted hASCs *in vivo*. Consistent with the results, overexpression of periostin in MSCs enhanced cell adhesion, survival rates, and cell adhesion-related signaling *in vitro* [35]. Implantation of periostin-overexpressed MSCs into infarcted myocardium resulted in enhanced survival of MSCs and directly prevented apoptosis of cardiomyocytes through a periostin-mediated paracrine mechanism. In addition, periostin induced proliferation of differentiated mammalian cardiomyocytes and promoted cardiac repair [19]. Periostin-stimulated proliferation of cardiomyocytes resulted in improved ventricular remodeling and myocardial function, reduced fibrosis and infarct size, and increased angiogenesis. Accumulating evidence suggests that periostin can inhibit apoptosis *via* inactivation of caspase and PARP cleavage and activation of pro-survival signaling [19, 36, 37]. Taken together with the current study, these results suggest that periostin has a beneficial effect on hASC-mediated bone healing by stimulating engraftment of transplanted hASCs.

Autologous implantation of BMSCs attached to HA/TCP increased cranial reconstruction in a canine animal model [38]. In addition, in the jaw defects, MSC-loaded HA/TCP scaffold induced more bone formation than HA/TCP scaffolds alone [39]. However, in the current study, using quantification analysis with micro-CT, we showed that HA/TCP scaffold loaded with either hASCs or periostin slightly, but not significantly, promoted bone formation compared with the control group, which received implantation with HA/TCP scaffold alone. Co-delivery of periostin with hASCs accelerated bone healing and formation of connective tissues compared with control groups transplanted with either hASCs or periostin alone. It was recently reported that periostin secreted by MSCs supports tendon formation in an ectopic mouse model. These results suggest that periostin may promote hASC-mediated bone healing by stimulating differentiation potential of hASCs. However, recombinant periostin did not stimulate osteogenic differentiation potential of hASCs *in vitro* (S2 Fig.), suggesting that osteogenic differentiation potential of hASCs may not be directly stimulated by periostin *in vivo*. Therefore, it is likely that periostin-stimulated survival and proliferation of transplanted hASCs may be responsible for the bone healing accelerated by co-implantation of hASCs and periostin. In addition, periostin has been reported to stimulate bone formation through a Wnt/β-catenin-dependent mechanism [40]. Therefore, it is possible to suggest that periostin promotes bone healing by stimulating proliferation of transplanted hASCs or by regulating Wnt/β-catenin signaling within bone tissues.

Angiogenesis is beneficial for treatment of critical sized bone defects by forming ectopic and vascularized bone [41]. hASCs have been reported to promote tissue repair by stimulating angiogenesis through a paracrine mechanism [42, 43]. hASCs secrete various angiogenic cytokines, proteases, protease inhibitors, and inflammatory mediators [44]. Vascular endothelial growth factor gene-activated matrix (VEGF_165_-GAM) enhances osteogenesis and angiogenesis in large segmental bone defects [45]. These results raised the possibility that periostin promotes angiogenic potential by stimulating secretion of angiogenic cytokines, such as VEGF, from hASCs. The successful achievement of stem cell-based bone regeneration depends on whether the transplanted stem cells can differentiate into functional osteogenic cells and whether those cells can produce paracrine factors stimulating proliferation of the tissue-resident cells within defective area. However, poor survival and engraftment efficiency of transplanted hASCs need to be solved for successful cell therapy. In our *in vitro* experiments, we found that recombinant periostin did not directly stimulate secretion of angiogenic cytokines, such as VEGF and IL-8, from hASCs (data not shown), although co-implantation of hASCs with periostin stimulated *in vivo* angiogenesis within calvarial defects. We demonstrate that recombinant periostin significantly improved the engraftment of transplanted hASCs *in vivo*. The percentages of PCNA-positive hASCs exhibited about 50% in the experimental group transplanted with hASCs and periostin 2 weeks after transplantation. At 8 weeks post-implantation, CM-DiI-positive hASCs were hardly detected (data not shown), suggesting eventual death of transplanted hASCs, although the percentage of Ki67-positive proliferating cells, including osteocytes and leukocytes, was estimated to approximately 50% (Fig. 5B). Therefore, it is likely that periostin promotes repair of defected bone tissues by stimulating survival of transplanted hASCs *in vivo*. In addition, our results showed that administration of periostin protein stimulated angiogenesis in calvarial defects. Consistently, recombinant periostin stimulated migration and tube formation of human endothelial progenitor cells *in vitro* and promoted blood perfusion in a murine hindlimb ischemia model [33]. Taken together, these results suggest that both periostin and hASCs are beneficial for healing of critical sized bone defects and co-delivery of periostin and hASCs synergistically promote bone repair by stimulating angiogenesis *in vivo*.

In summary, the present study demonstrates that periostin stimulates survival and bone healing capacity of transplanted hASCs in a murine calvarial defect model. Co-delivery of recombinant periostin together with hASCs will be useful for cell therapy of injured tissues, including critical sized bone defects. With further pre-clinical optimization, this approach may improve the outcome of current cell therapy for calvarial defects.

## Supporting Information

S1 Fig Effects of hASCs and periostin on proliferation of osteocytes and leukocytes in calvarial defects. Double immunofluorescence staining of anti-Ki67 antibody (proliferating cell marker) with antibodies against either osteocalcin (osteocyte marker) or CD45 (leukocyte marker). Overlaid images of Ki67 (green color), nuclei (DAPI, blue color), and osteocalcin (red color) or CD45 (red color) are shown. Scale bar = 20 μm
(TIF)

S2 Fig Effect of periostin on osteogenic differentiation of hASCs. hASCs were incubated in growth medium or osteogenic differentiation medium (10% FBS, 0.1 μM dexamethasone, 10 mM β-glycerophosphate, and 50 μM ascorbic acid in α-minimum essential medium) in the absence (-) or presence (+) of recombinant periostin (10 μg/mL) over 2 weeks. Extracellular matrix calcification was visualized by Alizarin Red S staining.
(TIF)
