# Supplementary figures and images for: Periostin Accelerates Bone Healing Mediated by Human Mesenchymal Stem Cell-Embedded Hydroxyapatite/Tricalcium Phosphate Scaffold
Source: PLoS One. 2015 Mar 16;10(3):e0116698. doi: 10.1371/journal.pone.0116698 (PMC4361583; doi:10.1371/journal.pone.0116698)

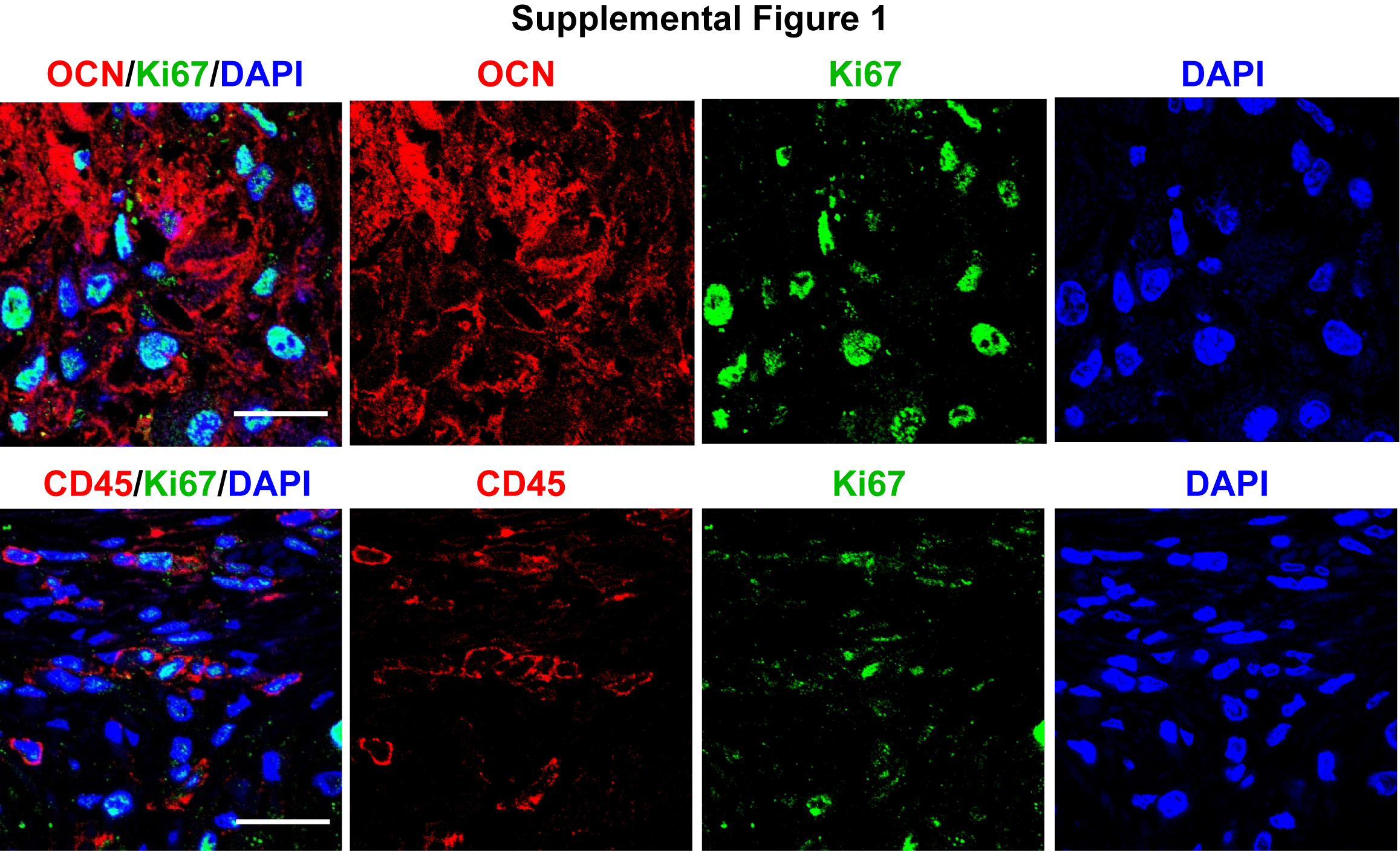

Supplement: S1 Fig — Double immunofluorescence staining of anti-Ki67 antibody (proliferating cell marker) with antibodies against either osteocalcin (osteocyte marker) or CD45 (leukocyte marker). Overlaid images of Ki67 (green color), nuclei (DAPI, blue color), and osteocalcin (red color) or CD45 (red color) are shown. Scale bar = 20 μm (TIF) [file pone.0116698.s001.tif]

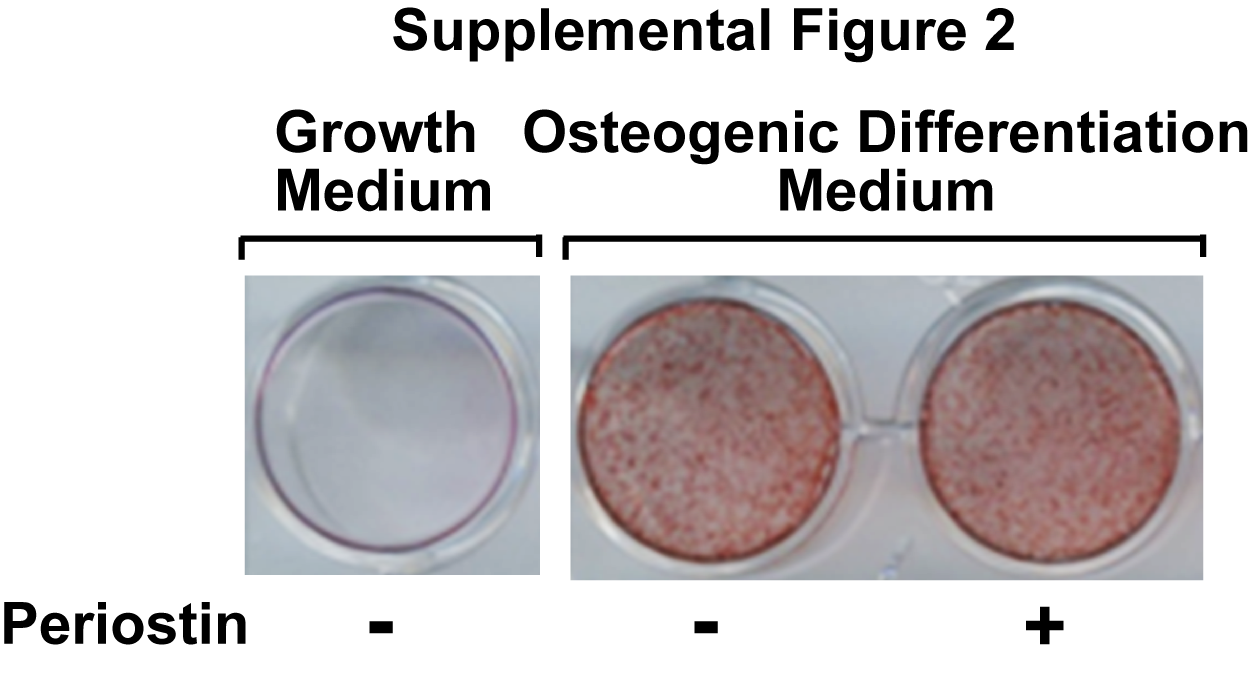

Supplement: S2 Fig — hASCs were incubated in growth medium or osteogenic differentiation medium (10% FBS, 0.1 μM dexamethasone, 10 mM β-glycerophosphate, and 50 μM ascorbic acid in α-minimum essential medium) in the absence (-) or presence (+) of recombinant periostin (10 μg/mL) over 2 weeks. Extracellular matrix calcification was visualized by Alizarin Red S staining. (TIF) [file pone.0116698.s002.tif]
